# Cadmium-induced apoptosis of Siberian tiger fibroblasts via disrupted intracellular homeostasis

**DOI:** 10.1186/s40659-016-0103-6

**Published:** 2016-10-24

**Authors:** Hui Wang, Zheng Liu, Wenxiu Zhang, Ziao Yuan, Hongyi Yuan, Xueting Liu, Chunwen Yang, Weijun Guan

**Affiliations:** 1 Jinzhou Medical University, Jinzhou, 121001 China; 2 Institute of Animal Science, Chinese Academy of Agricultural Sciences, Beijing, 100193 China; 3 College of Life Science and Technology, Mudanjiang Normal University, Mudanjiang, 157012 China

**Keywords:** Siberian tiger, Cadmium, Apoptosis, Fibroblast, Calcium

## Abstract

**Background:**

Heavy metals can cause great harm to Siberian tigers in the natural environment. Cadmium (Cd^2+^) is an environmental contaminant that affects multiple cellular processes, including cell proliferation, differentiation, and survival. It has been shown to induce apoptosis in a variety of cell types and tissues.

**Results:**

We investigated the apoptotic effects of Cd^2+^ on Siberian tiger fibroblasts in vitro. Our research revealed the typical signs of apoptosis after Cd^2+^ exposure. Apoptosis was dose- (0–4.8 μM) and duration-dependent (12–48 h), and proliferation was strongly inhibited. Cd^2+^ increased the activity of caspase-3, -8, and -9 and disrupted calcium homeostasis by causing oxidative stress and mitochondrial dysfunction. It also increased K^+^ efflux and altered the mRNA levels of Bax, Bcl-2, caspase-3, caspase-8, Fas, and p53.

**Conclusions:**

Our results suggest that Cd^2+^ triggers the apoptosis of Siberian tiger fibroblasts by disturbing intracellular homeostasis. These results will aid in our understanding of the effects of Cd^2+^ on Siberian tigers and in developing interventions to treat and prevent cadmium poisoning.

## Background

The tiger (*Panthera tigris*) ranges only in Asia [1]. There are four recognized subspecies of tigers in China, namely, the Siberian tiger (*P. t. altaica*) in northeast China, the Indochinese tiger (*P. t. corbetti*) in Yunnan, the South China tiger (*P. t. amoyensis*) in the middle east and south of China, and the India or Bengal tiger (*P. t. tigris*) in the Tibetan region. Currently, it is estimated that there are less than 400 Siberian tigers in eastern Russia, northeastern China, and Korea, with less than 20 ranging specifically in China [2, 3]. The remaining wild tiger population continues to shrink in China in response to increasing pressure from human-related factors. Moreover, the diversity of the tiger’s available genetic resources has gradually decreased. Habitat degradation is one reason why the tiger is on the edge of extinction and has become one of the most endangered large cat species in the world. With the rapid development of modern industry, agriculture, and transportation, the tiger habitat is under serious threat. It is especially threatened by cadmium (Cd^2+^) pollution, as this metal can cause great harm to the growth and development of tigers.

Cd^2+^ exists in the earth’s crust and is a naturally occurring heavy metal. It is an environmental and industrial pollutant that has increased in abundance worldwide, causing significant ecological problems. It is toxic even at low doses partly owing to its long biological half-life after ingestion or inhalation. Long-term exposure to Cd^2+^ causes a variety of pathological conditions and poisons many cell types, including kidney, liver, brain, testis, lung, and thymus, both in vitro and in vivo [4–6]. Cd^2+^ compounds promote tumorigenesis in the testes, lungs, and prostate as well as hematopoietic malignancies [7]. They also morphologically transform and induce chromosomal aberrations and gene mutations in cultured mammalian cells [8].

Cd^2+^ affects multiple cellular events, including proliferation, differentiation, and survival. It frequently alters the activity of biological molecules by binding to nitrogen, oxygen, or sulfur-containing groups, eventually disrupting normal cell function [9, 10]. Multiple lines of evidence indicate that apoptosis plays an important role in acute and chronic Cd^2+^ intoxication. Cd^2+^ has been shown to induce apoptosis, as indicated by cell contraction, annexin V overexpression, reactive oxygen species (ROS) generation, DNA fragmentation, and cell cycle arrest, in a dose-dependent manner [11, 12].

In the environment, Siberian tigers are exposed to Cd^2+^ via the consumption of contaminated food, water, and metal-smoldering fumes, which accounts in part for their dwindling numbers [13]. While Cd^2+^-induced apoptosis in Siberian tiger fibroblasts and other mammalian cell types has been documented, its underlying mechanisms remain unknown [14, 15]. Therefore, we aimed to further examine this process in Siberian tiger fibroblasts and to identify the responsible cellular and molecular mechanisms. We specifically evaluated the effects of Cd^2+^ on cell proliferation, mitochondrial function, calcium (Ca^2+^) homeostasis, caspase activation, and gene expression. We also established criteria for the early diagnosis of Cd^2+^ poisoning in Siberian tigers and the safety threshold for this metal. Lastly, we provide theoretical scientific and experimental information that will help protect Siberian tigers against Cd^2+^ poisoning.

## Results

### Growth dynamics

**Fig. 1:**
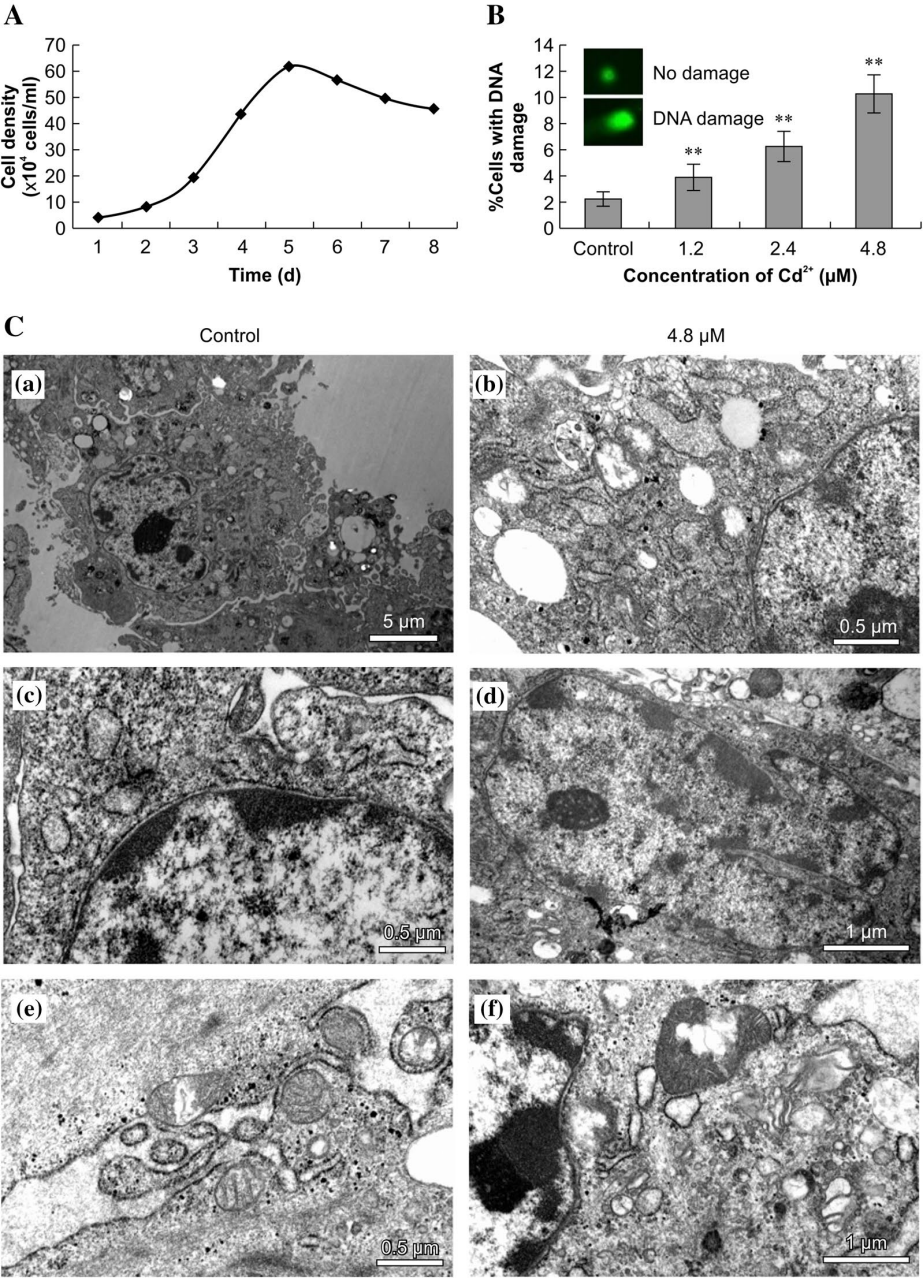
Comet assays and transmission electron microscopy (TEM). **A** Siberian tiger fibroblast growth curve. **B** Comet assays show DNA damage in Siberian tiger fibroblasts treated with cadmium (Cd^2+^) for 24 h. Statistically significant differences compared to the control cells are denoted by a *double asterisk* (P < 0.01; n = 3). **C** TEM shows disruption of the subcellular architecture in Siberian tiger fibroblasts treated with Cd^2+^ for 24 h. *a*, *c*, *e* control cells. *b*, *d*, *f* Cd2+ -treated cells. The *scale bars* are 5 μm (*a*), 0.5 μm (*b*, *c*, *e*), and 1 μm (*d*, *f*)

The Siberian tiger fibroblast growth curve showed a clear “S” shape, and the population doubling time (PDT) was 32 h (Fig. 1A). Cells entered the logarithmic growth phase 1–2 days after seeding and grew logarithmically from days 2–5. Cell density peaked on days 5 and 6 and subsequently declined.

### Comet assay

Control (no Cd^2+^) cells had intact nuclear DNA. In contrast, Cd^2+^-treated cells had DNA strand breaks, as well as DNA fragments that migrated along the electric field of the bright fluorescent tail in the comet assay (Fig. 1B). The size of the tail (which correlates with the severity of the damage) increased as a function of Cd^2+^ dose. These results indicate that Cd^2+^ undermines the integrity of the DNA of Siberian tiger fibroblasts. The comet assay has been shown to be a sensitive means of detecting nucleosome DNA fragmentation at the single-cell level [16].

### Transmission electron microscopy (TEM)

Apoptosis is indicated by chromatin condensation, nuclear fragmentation, and nuclear and cytoplasmic blistering. The control cells had plump cytoplasm, as well as intact nuclear membranes and mitochondrial structures, whereas the Cd^2+^-treated cells contained small vacuoles, dense chromatin masses, and karyopyknotic nuclei (Fig. 1C). Moreover, the electron density of the cytoplasm of the treated cells was increased, indicating marginalization, and the nuclear membranes had begun to decompose.

### Annexin V-fluorescein isothiocyanate (FITC)/propidium (PI) double-labeling

**Fig. 2:**
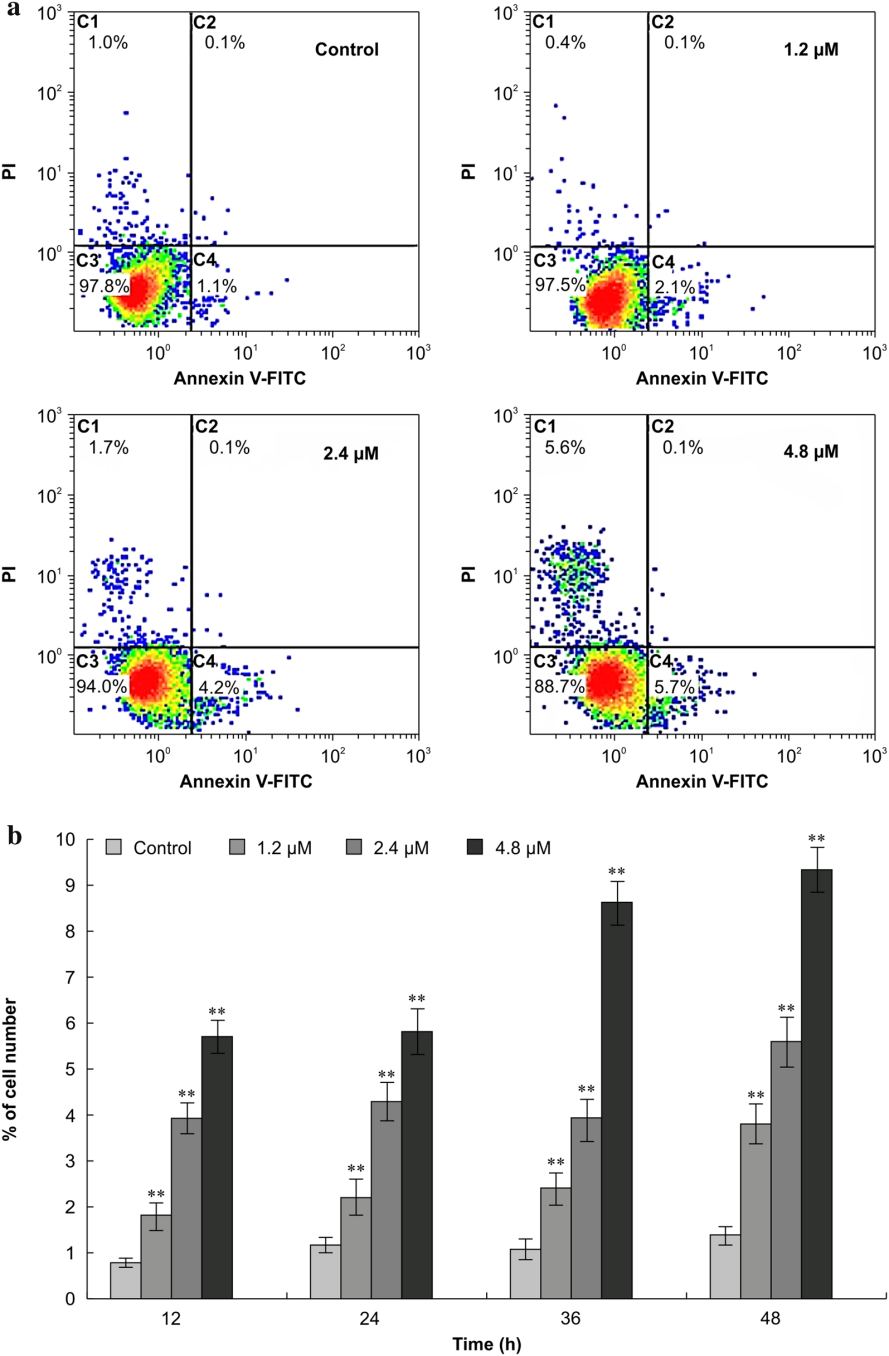
Annexin V-fluorescein isothiocyanate/propidium iodide double-labeling. **a** Percentage of stained Siberian tiger fibroblasts in the presence (24 h) or absence of cadmium (Cd^2+^). **b** Apoptotic rates of Siberian tiger fibroblasts treated with Cd^2+^ for 12, 24, 36 and 48 h. Statistically significant differences compared to the corresponding controls are denoted by a *double asterisk* (P < 0.01; n = 3)

Apoptosis was quantified by measuring annexin V-FITC and PI staining (which indicates phosphatidylserine externalization) via flow cytometry. Cd^2+^ increased the percentage of stained cells, and within the dose range and time frame used in this experiment, this response was both dose- and time-dependent (Fig. 2). The apoptosis rate was significantly higher in the treated cells than in the control cells (P < 0.01). Moreover, 4.8 μM Cd^2+^ significantly increased the percentage of necrotic cells at 24, 36, and 48 h although not at 12 h (data not shown).

### Cell cycle analysis

**Fig. 3:**
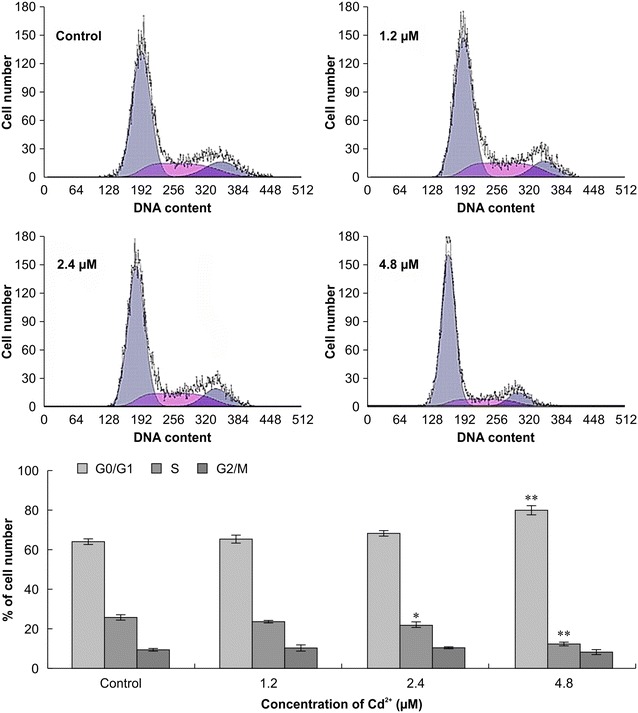
Cell cycle progression of Siberian tiger fibroblasts treated with cadmium for 24 h. The distribution of cells in the G0/G1, S, and G2/M phases of the cell cycle was determined via flow cytometry. Statistically significant differences compared with the corresponding controls are denoted by an *asterisk* (P < 0.05) and a *double asterisk* (P < 0.01; n = 3)

To determine the mechanism underlying the toxic effect of Cd^2+^ on Siberian tiger fibroblasts, we analyzed cell cycle progression via flow cytometry. We found that the percentage of cells in the G0/G1 phase of the cell cycle increased, and the percentage of cells in the S and G2 phases correspondingly decreased, as Cd^2+^ concentration increased (Fig. 3). These results show a dose–response relationship between Cd^2+^ concentration and cell cycle perturbation.

### Mitochondrial membrane potential

**Fig. 4:**
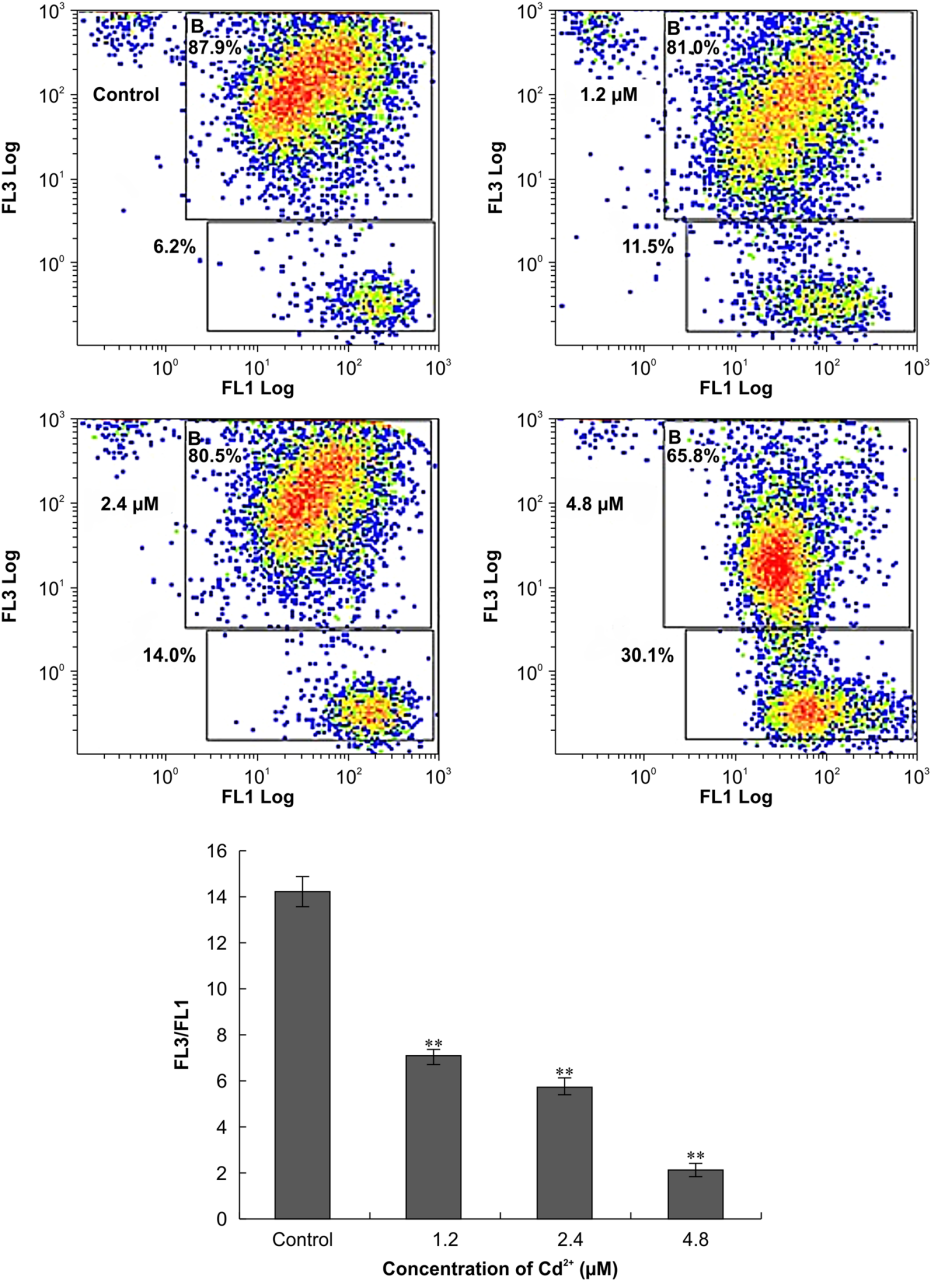
Mitochondrial membrane potential of Siberian tiger fibroblasts treated with cadmium for 24 h. Statistically significant differences compared with the control are denoted by a *double asterisk* (P < 0.01; n = 3)

5,5′, 6,6′-tetrachloro-1,1′,3,3′-tetraethylbenzimidazolcarbocyanine iodide (JC-1) is a lipophilic cationic dye that is selectively taken up by mitochondria. Its color changes from green to red as the membrane potential increases. In normal cells, JC-1 aggregates in the mitochondria and fluoresces red. In apoptotic or necrotic cells, JC-1 is monomeric and stains the cytoplasm green. Thus, the number of C-gated cells reflects the mitochondrial membrane potential, and a greater number indicates a reduction in mitochondrial membrane potential. Cells treated with Cd^2+^ had a significantly lower mitochondrial membrane potential than did control cells (P < 0.01; Fig. 4).

### Intracellular Ca^2+^ homeostasis

**Fig. 5:**
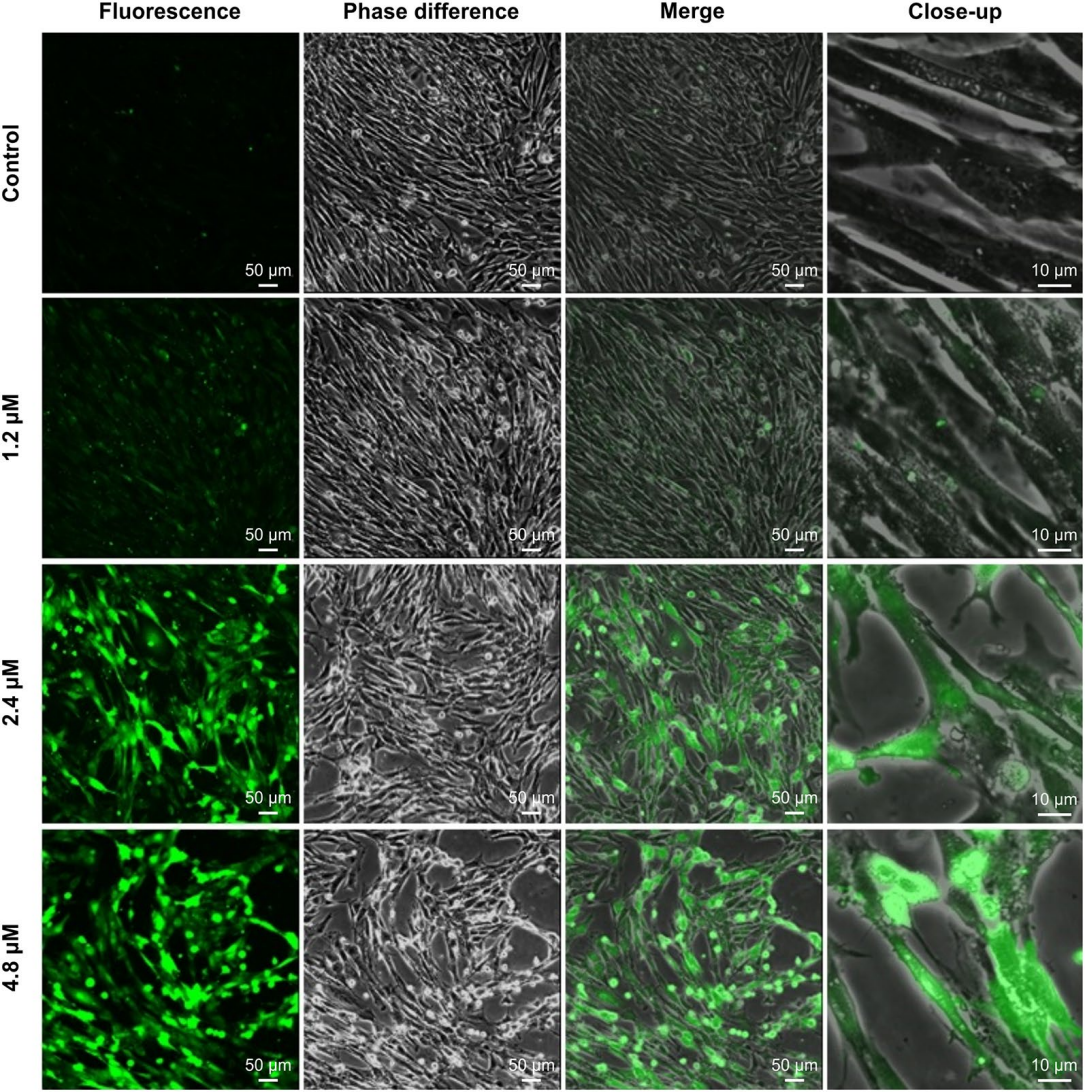
Effects of cadmium on intracellular calcium homoeostasis in Siberian tiger fibroblasts. Cells received Cd^2+^ for 24 h. The fluorescence, phase difference, and merged image *scale bars* are 50 μm. The close-up image *scale bar* is 10 μm

Our results show that increases in Cd^2+^ concentration progressively reduce the mitochondrial membrane potential in Siberian tiger fibroblasts (i.e., increase the green/red fluorescence ratio), which is indicative of apoptosis. Decreases in mitochondrial membrane potential are caused by increases in the free Ca^2+^ concentration [17]. The green fluorescence seen in cells treated with 2.4 or 4.8 μM Cd^2+^ was widely distributed, indicating that intracellular calcium homeostasis had been severely disrupted (Fig. 5).

### ROS analysis

**Fig. 6:**
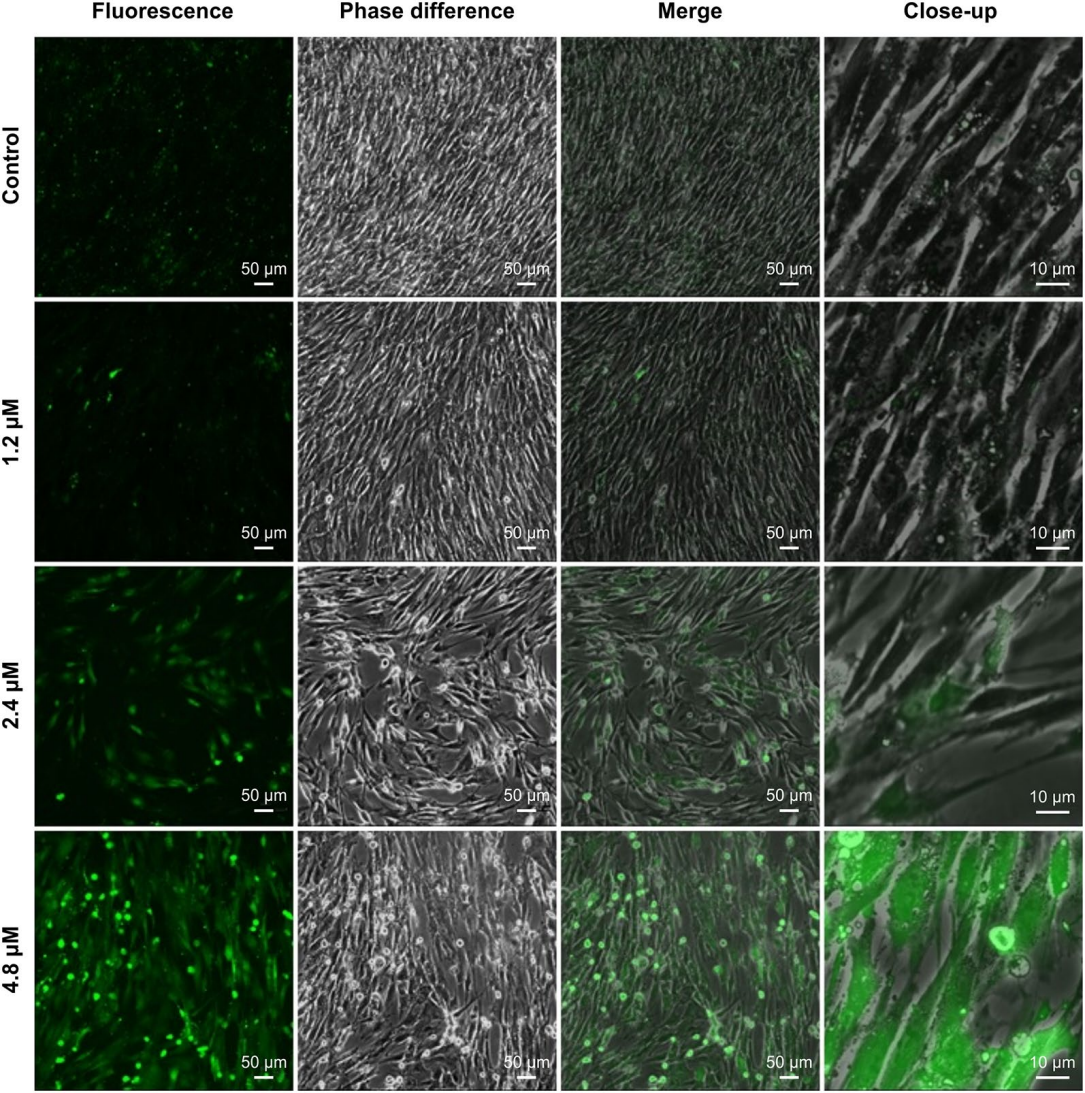
Effects of cadmium (Cd^2+^) on intracellular reactive oxygen species levels in Siberian tiger fibroblasts. Cells received Cd^2+^ for 24 h. The fluorescence, phase difference, and merge image *scale bars* are 50 μm. The close-up image *scale bar* is 10 μm

ROS are subcellular messengers that play a role in several cellular processes, including apoptosis. We used ROS green fluorescence probes with 2′,7′- dichlorodihydrofluorescein-diacetate (DCFH-DA) detection to identify apoptotic cells via confocal microscopy. We found an increase in ROS levels, as indicated by a more intense green fluorescence signal, in cells receiving the higher Cd^2+^ concentrations (Fig. 6). Moreover, the green fluorescence that resulted from 4.8 μM Cd^2+^ treatment was distributed throughout the cell.

### Extracellular Ca^2+^ and potassium (K^+^) flux measurement

**Fig. 7:**
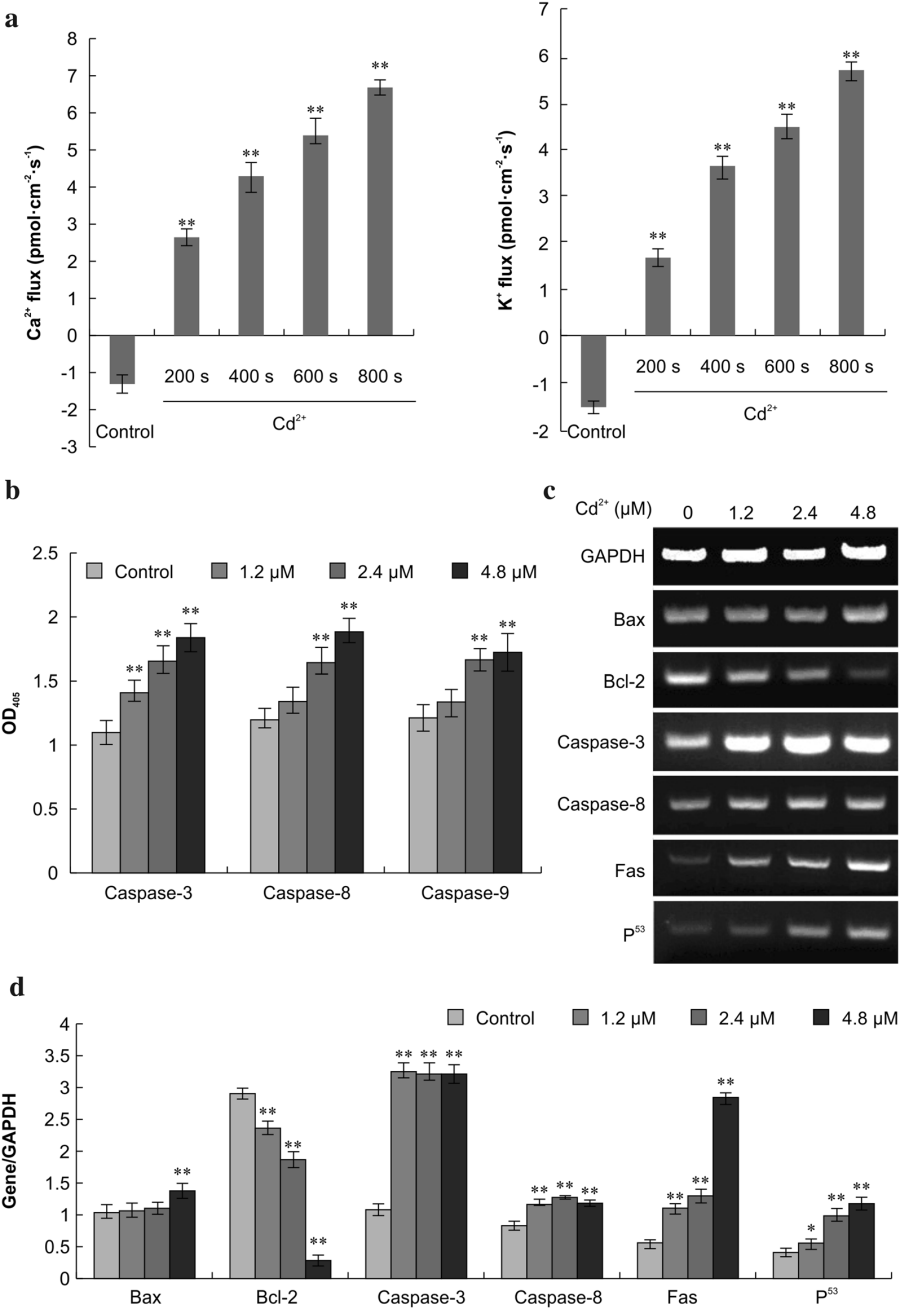
Calcium (Ca^2+^) and potassium (K^+^) flux, caspase activity, and gene expression. **a** Ca^2+^ and K^+^ flux was measured before and after treatment of Siberian tiger fibroblasts with 4.8 μM Cd^2+^ by using the ion flux estimate technique. Statistically significant differences compared to the corresponding controls are denoted by an *asterisk* (P < 0.05) and a *double asterisk* (P < 0.01; n = 3). **b** Activities of caspase-3, -8, and -9 were determined in cytosolic extracts of Siberian tiger fibroblasts. **c**, **d** Expression levels of the indicated mRNAs were determined via reverse transcription-polymerase chain reaction 24 h after exposure to Cd^2+^ and normalized to glyceraldehyde-3-phosphate dehydrogenase mRNA levels

Using the microelectrode ion flux estimation (MIFE) technique, we assessed the size and direction of Ca^2+^ and K^+^ flow (vertical axis) over time (horizontal axis) in control and Cd^2+^-treated Siberian tiger fibroblasts. The abscissa (positive) on the top curve represents Ca^2+^ and K^+^ outflow, while the abscissa on the lower curve represents Ca^2+^ and K^+^ inflow (Fig. 7a). Experimental results showed that Ca^2+^ influx mainly occurred after Cd^2+^ addition (0–200 s), whereas K^+^ influx was apparent before addition (0–200 s). Both Ca^2+^ and K^+^ efflux increased after Cd^2+^ addition (200–800 s), and the flow rate tended to increase over time. There were significant differences in Ca^2+^ and K^+^ flux before and after treatment.

### Caspase-3, -8, and -9 activity

Caspase-3, -8, and -9 are reactive proteases that play important roles in the apoptotic process. We evaluated their activity by using a spectrophotometer to detect cleaved substrates; higher optical density values indicate greater caspase activity. Caspase-3 activity was more pronounced in cells in cells receiving 1.2, 2.4, or 4.8 μM Cd^2+^ than in control cells (P < 0.01, Fig. 7b). Cd^2+^ also markedly increased the activity of caspase-8 and -9 at 2.4 and 4.8 μM (P < 0.01), although not at 1.2 μM. Collectively, these results indicate that Cd^2+^ activates caspase-3, -8, and -9 in Siberian tiger fibroblasts.

### Reverse transcription polymerase chain reaction (RT-PCR)

Bax, Bcl-2, caspase-3, caspase-8, Fas, and p53 mRNA expression levels were determined via RT-PCR. Bcl-2 and activated Bax reside in the outer mitochondrial membrane, and the Bax/Bcl-2 ratio determines whether apoptosis will be inhibited or promoted. RT-PCR analysis showed that Cd^2+^ significantly increased the expression of Bax mRNA, as well as that of caspase-3, caspase-8, Fas, and p53 mRNA (P < 0.05 in all cases). Cd^2+^ also significantly reduced Bcl-2 mRNA levels (P < 0.01; Fig. 7c, d). Thus, exposure to Cd^2+^ may increase the Bax/Bcl-2 protein ratio in Siberian tiger fibroblasts.

## Discussion

Cd^2+^ is a ubiquitous toxic metal that exerts genotoxic effects and induces apoptosis in many cell types [18, 19]. In vitro studies have shown that low concentrations of Cd^2+^ trigger apoptosis, while higher concentrations induce necrosis [20]. Cd^2+^ exposure can also inhibit the generation of mitochondrial ATP, leading to oxidative stress, caspase activation, and inappropriate gene expression [21]. Long-term exposure to Cd^2+^ resulted in emphysema in rat lung in vivo models [22].

Our previous study suggested that Cd^2+^ induced apoptosis in Siberian tiger fibroblasts by inhibiting DNA synthesis, reducing mitochondrial membrane potential, and interfering with Ca^2+^ homeostasis [15]. In this study, we evaluated the impact of Cd^2+^ toxicity on Siberian tiger fibroblasts, specifically assessing how the metal affected cell function and how it induced changes in cell morphology. We evaluated Cd^2+^-induced damage to subcellular structures, cell atrophy, mitochondrial depolarization, and chromatin condensation. Cd^2+^-induced apoptosis led to phosphatidylserine translocation from the inner cell membrane to the surface of the cell, and this effect was dose- and time-dependent in the range of 0–4.8 μM Cd^2+^ over 12–48 h. High concentrations of Cd^2+^ did not further increase apoptosis, but instead increased necrosis.

Physiological and pathological apoptotic stimuli affect cell cycle progression [23], and cell cycle arrest can induce apoptosis. Thus, analysis of the cell cycle is one of the most important measurements of apoptotic activity [24]. Our data showed that increasing concentrations of Cd^2+^ increased the proportion of cells in G0/G1 and reduced the proportion cells in S phase. This finding indicates that exposure of Siberian fibroblasts to Cd^2+^ promotes G0/G1 arrest and consequently precludes S phase entry and cell proliferation. Therefore, we speculate that the destructive effects of Cd^2+^ on Siberian tiger fibroblasts are due to inhibition of DNA replication and cell cycle progression, resulting in Siberian tiger fibroblast apoptosis. This is an important finding that will contribute to future research aimed at understanding the mechanisms of Cd^2+^-induced apoptosis in tiger fibroblast cells.

Our findings suggest that Cd^2+^-induced apoptosis in Siberian tiger fibroblasts results from activation of caspase-3, -8, and -9 and thus via a caspase-dependent pathway. Both intrinsic (caspase-9-mediated) and extrinsic (caspase-8-mediated) apoptotic pathways have been shown to trigger caspase activation in cells undergoing apoptosis [25, 26].

Cd^2+^ exposure indirectly causes oxidative stress, namely by reducing the intracellular levels of antioxidants and by increasing the generation of ROS via mitochondrial dysfunction [27, 28]. Excess ROS can have serious impacts on mitochondria, leading to oxidative damage, which promotes steroidogenesis in the liver and kidney. We previously found that exposure to Cd^2+^ at low levels for 90 days disrupted the balance between ROS production and the antioxidant defense [29]. Here we show that, at certain concentrations, Cd^2+^ increases ROS levels, destroying the cell oxidant-antioxidant balance. Such an imbalance could result in oxidative damage, and we suggest that oxidative damage is one of the underlying mechanisms of Cd^2+^-induced apoptosis in Siberian tiger fibroblasts.

A drop in the mitochondrial membrane potential below a certain threshold is an early event in apoptosis. The current study showed decreases in the mitochondrial membrane potential of Siberian tiger fibroblasts with increasing concentrations of Cd^2+^. This finding suggests that mitochondria may be a critical target of Cd^2+^ cytotoxicity, since a disruption in the electrochemical gradient of the mitochondrial membrane may initiate the apoptotic pathway [30, 31]. However, the specific toxic effects of Cd^2+^ on mitochondria and the relative sensitivity of different mitochondrial sites to the processing of Cd^2+^ are not well understood.

Ca^2+^ as a second messenger is necessary for cell metabolism, and it is well accepted that a disruption in Ca^2+^ homeostasis can induce apoptosis in multiple ways. Our data showed that Cd^2+^ exposure increased free Ca^2+^ levels in the cytoplasm, allowing intracellular Ca^2+^ homeostasis to be disturbed. This homeostatic disruption likely led to the drop in mitochondrial membrane potential, which could have caused further release of Ca^2+^ from the endoplasmic reticulum. Previous studies identify the sources of free intracellular Ca^2+^ levels as Ca^2+^ released from intracellular stores and Ca^2+^ imported from outside the cell. Here, we found that Ca^2+^ flowed into the cell before Cd^2+^ treatment, but exited after treatment. This finding suggests that the observed increase in free intracellular Ca^2+^ levels was due to the release of endogenous Ca^2+^ stores, rather than the absorption of extracellular Ca^2+^. Further investigations of the role of Ca^2+^ in Cd^2+^-induced apoptosis will be important for a full understanding of the mechanisms of Cd^2+^ toxicity.

Similar to cell shrinkage, nuclear condensation, DNA breakage, and formation of apoptotic bodies, the loss of K^+^ is an early characteristic of apoptosis [32]. The loss of intracellular K^+^ results in an intracellular hypotonic state and accounts in part for shrinkage of the cell body [33]. Our results showed K^+^ influx before Cd^2+^ exposure and efflux after exposure. Further, we observed that the K^+^ flow rate exhibited an increasing trend with the extension of treatment time.

The Bcl-2 family of proteins consists of both pro-apoptotic members, such as Bax, and anti-apoptotic members, such as Bcl-2. The ratio of anti-apoptotic to apoptotic forms appears to dictate the response to a death stimulus, although both Bcl-2 and Bax can regulate apoptosis independently of one another. Bax and Bcl-2 expression has been reported in a variety of cell types, and it appears that Cd^2+^ treatment is associated with apoptosis [34]. Caspase-8 is considered to be an upstream (initiator) caspase because it interacts with adaptor proteins following activation of cell-surface death receptors [35]. The addition of cytochrome c to Jurkat cell-free extracts initiates a cascade of protease activation in vitro that involves caspase-3, -8, and -9 [36]. The death receptor Fas also plays a role in mediating apoptosis, with an up-regulation of this molecule enhancing signal transduction in the cell death cascade [37, 38]. Finally, p53 is known to execute mitochondria-mediated apoptosis via transcription-dependent and -independent pathways [39]. Activation of the p53 pathway is required for apoptosis induction by growth factor withdrawal, hypoxia, and DNA damage [40, 41].

While the specific association between the abovementioned molecules and Cd^2+^ in Siberian tiger fibroblasts is unknown, the present results show an upregulation of p53 mRNA following Cd^2+^ exposure. Further, we observed that Cd^2+^ reduced the mRNA expression of Bcl-2, but not of Bax, caspase-3, caspase-8, or Fas, in a dose-dependent manner. Thus, the exposure of Siberian tiger fibroblast to Cd^2+^ greatly increased the Bax/Bcl-2 mRNA ratio. It should be noted that a previous study showed that Cd^2+^-induced apoptosis involves mitochondrial stress due to the downregulation of anti-apoptotic factors [42]. However, suppression of Bcl-2 gene expression is a complex phenomenon, which involves inactivation of transcription factors or their inability to bind the Bcl-2 gene promoter.

## Conclusion

In short, exposure of Siberian tiger fibroblasts to Cd^2+^ significantly increased cytotoxicity, apoptosis, and necrosis. These events were associated with inhibition of DNA synthesis, Ca^2+^ overload, oxidative stress, K^+^ outflow, gene expression, and mitochondrial dysfunction. Our study shows that Cd^2+^ treatment can induce apoptosis via the mitochondrial pathway and disrupt intracellular homeostasis in Siberian tiger fibroblasts. These results provide insight into the mechanisms of Cd^2+^-apoptosis, as well as the adverse effects of Cd^2+^ on the Siberian tiger. Although the results of our study offer useful information for conserving the Siberian tiger population, we note that in vitro experimental systems do not fully recapitulate the mechanisms of Cd^2+^-induced apoptosis in living organisms.

## Methods

### Materials

Siberian tiger fibroblasts were provided by the Institute of Animal Science at the Chinese Academy of Agricultural Sciences. They were authenticated by the supplier and tested for contamination, with negative results. PI and cadmium chloride (CdCl_2_) were purchased from Sigma-Aldrich (St. Louis, MO, USA). Dulbecco’s modified Eagle’s medium (DMEM) and fetal bovine serum (FBS) were purchased from Gibco/BRL (Grand Island, NY, USA). The annexin V-FITC Apoptosis Detection Kit I was purchased from Becton, Dickinson and Company (Franklin Lakes, NJ, USA).

### Cell culture

Cells were cultured in DMEM supplemented with 10 % FBS at 37 °C under humidified conditions with 5 % CO_2_ and 95 % air in the atmosphere. The medium was refreshed every other day. Cells were detached from the flasks and split 1:1 when they reached 80–90 % confluence.

### Growth dynamics

Suspended cells (1 ml) were seeded into 24-well microplates at a concentration of 2 × 10^4^ cells ml^−1^. The growth rate was determined by using a hemocytometer; the number of cells in three grids was determined each day for 8 days. A growth curve was drawn, and the PDT was calculated based on the curve.

### Drug solution preparation and cell treatment

Cd^2+^ was supplied to cells as CdCl_2_, which was dissolved in DMEM and sterilized by filtration. Logarithmically growing cells were treated with a series of Cd^2+^ concentrations (0, 1.2, 2.4, or 4.8 μM) for predetermined times [43, 44].

### Comet assay

Using ice-cold phosphate-buffered saline (PBS), cells were washed and suspended at a concentration of 1 × 10^5^ cells ml^−1^. The comet assay was performed by using the Alkaline Comet Assay kit (Trevigen Inc., Gaithersburg, MD, USA) as per the manufacturer’s instructions. Briefly, the cell suspension was mixed with low melting point-agarose, chilled, and stored in ice-cold lysis buffer (Trevigen Inc.) at 4 °C. The comet slides were immersed sequentially in alkaline electrophoresis buffer and 70 % ethanol. Comet cell DNA was then stained with SYBR Green (Sigma-Aldrich), and cells were imaged by using a confocal microscope (TE-2000-E; Nikon, Tokyo, Japan).

### TEM

Cells were washed twice in PBS and fixed with 2.5 % (m v^−1^) glutaraldehyde followed by 1 % osmium tetroxide. They were then embedded in epoxy resin and acetone, and the samples were allowed to solidify after being dehydrated through a gradient of ethanol concentrations (30, 50, 70, 80, 90 and 100 %). Ultrathin Sects. (50 nm) were prepared with uranyl acetate and lead citrate for TEM (JEM-2000EX; JEOL Ltd., Tokyo, Japan).

### Annexin V-FITC/PI double-labeling

The annexin V-FITC Apoptosis Detection Kit I was used in accordance with the manufacturer’s instructions. Cells (5 × 10^5^ ml^−1^ in 100 μl binding buffer) were incubated with 5 μl FITC and 5 μl PI for 15 min, and 400 μl binding buffer was added to each sample before analysis of the stained cells via flow cytometry (FC500; Beckman Coulter Corp., Brea, CA, USA). At least 10,000 cells per sample were analyzed. Experiments were performed in triplicate.

### Cell cycle analysis

Cells were suspended in ice-cold 70 % ethanol and kept at 4 °C overnight. Cells were then incubated with RNase A (0.02 mg ml^−1^) and stained with PI (PI 0.05 mg ml^−1^) in a buffer containing 0.585 g ml^−1^ NaCl and 1 mg ml^−1^ sodium citrate, pH 7.2–7.6) at 4 °C for 30 min before being run through the flow cytometer (FC500). A total of 10,000 cells were recorded for each sample, and the percentages of cells in the G0/G1, S, and G2/M phases of the cell cycle were plotted as DNA histograms.

### Mitochondrial membrane potential

Cells (1 × 10^6^ ml^−1^) were incubated with JC-1 (5 g ml^−1^, 0.5 ml sample^−1^) at 37 °C for 15 min and rinsed in JC-1 staining buffer. Each sample was resuspended in 0.5 ml of PBS and then immediately analyzed via flow cytometry (FC500). At least 10,000 cells were evaluated in each sample.

### Intracellular Ca^2+^ homeostasis

Cells (1–2 × 10^6^ ml^−1^) were loaded with 1-[2-amino-5-(2,7-dichloro-6-acetoxymethoxy-3-oxo-9-xanthenyl)phenoxy]-2-(2-amino-5-methylphenoxy)ethane-N,N,N’,N’-tetraacetic acid, tetra(acetoxymethyl)ester (Fluo3-AM; Invitrogen, Carlsbad, CA, USA) (final concentration 5–10 µM in dimethyl sulfoxide) and incubated at 37 °C in 5 % CO_2_ for 30–60 min in the dark. They were gently shaken a few times during incubation and then washed twice with DMEM. A negative control was also prepared (not Fluo3-AM-loaded), and cells were observed and photographed by using a confocal microscope (TE-2000-E).

### ROS analysis

Cells (1–2 × 10^6^ ml^−1^) were loaded with DCFH-DA (Molecular Probes, Eugene, OR, USA) at a final concentration of 10 μM. Cells were incubated at 37 °C in 5 % CO_2_ for 20 min, gently shaken a few times, and then washed twice with serum-free DMEM. Cells were observed and photographed via confocal microscopy (TE-2000-E).

### Extracellular Ca^2+^ and K^+^ flux measurement

The MIFE technique, which noninvasively measures Ca^2+^ and K^+^ fluxes, was carried out by using selective microelectrodes according to the methodology previously described for the surface measurement of a net flow of ions in dense cell monolayers [33, 45]. Briefly, cells (1 ml, 1 × 10^5^ ml^−1^) were seeded in petri dishes and treated with Cd^2+^ during the logarithmic growth phase. Before and after testing with each electrode, Ca^2+^ and K^+^ levels were determined by using calibration standards. Ion flux between two microelectrode positions—one close to the cell monolayer (5 µm) and one further away (up to 20 μm)—was measured as the electrochemical potential of Ca^2+^ and K^+^ at the two offset points.

### Caspase-3, -8, and -9 activity assay

The activities of caspase-3, -8, and -9 were evaluated by using the Colorimetric Assay kit (Pik-Day Biotechnology, Haimen, China). Protein concentrations of the supernatants of cell lysates (100 µl lysate obtained from 2 × 10^6^ cells) were determined using the Bradford Assay kit (Beyotime Biotechnology, Haimen, China). Protein-normalized supernatants (10 µl) were mixed with 10 µl of Ac-DEVD-pNA (2 mM) for caspase-3, Ac-IETD-pNA (2 mM) for caspase-8, or Ac-LEHD-pNA (2 mM) for caspase-9 in assay buffer. Activity was determined via spectrophotometric measurement of the reaction product (ND-1000; NanoDrop Technology, Wilmington, DE, USA).

### Reverse transcription-polymerase chain reaction (RT-PCR)

Total RNA was extracted from cells, and synthesis of the first cDNA strand was performed by using a PrimerScript TM RT kit (Takara, Dalian, China). The primer pairs for the genes encoding Bax, Bcl-2, caspase-3, caspase-8, Fas, p53, and glyceraldehyde-3-phosphate dehydrogenase (a housekeeping protein) were designed by using Premier 5.0 software (Premier Biosoft, Palo Alto, CA, USA) and synthesized by Shanghai Biological Technology Co., Ltd. (Shanghai, China). The PCR conditions were as follows: denaturation at 94 °C for 5 min, 30 cycles of 30 s each at 94 °C, 30 s of annealing, 72 °C for 30 s and a 72 °C extension for 10 min. PCR products were identified via electrophoreses on 1.5 % agarose gels.

### Statistical analysis

We repeated each experiment at least three times to confirm reproducibility. All values are expressed as mean ± standard deviation. Data were analyzed by using Statistical Analysis System software (SAS Institute Inc., Cary, NC, USA), and compared by using Duncan’s multiple comparison test. P < 0.05 was considered statistically significant.

## Abbreviations

Ca^2+^: calcium
Cd^2+^: cadmium
CdCl_2_: cadmium chloride
DCFH-DA: 2′, 7′-dichlorodihydrofluorescein-diacetate
DMEM: Dulbecco’s modified Eagle’s medium
FBS: fetal bovine serum
FITC: fluorescein isothiocyanate
JC-1: 5,5′, 6,6′-tetrachloro-1,1′,3,3′-tetraethylbenzimidazolcarbocyanine iodide
MIFE: microelectrode ion flux estimation
PBS: phosphate-buffered saline
PDT: population doubling time
PI: propidium iodide
ROS: reactive oxygen species
RT-PCR: reverse transcription polymerase chain reaction
TEM: transmission electron microscopy
